# (7-Chloro-2-oxo-2*H*-chromen-4-yl)methyl piperidine-1-carbodithio­ate

**DOI:** 10.1107/S1600536812007933

**Published:** 2012-02-29

**Authors:** K. Mahesh Kumar, Dalbir Kour, Kamini Kapoor, N. M. Mahabaleshwaraiah, O. Kotresh, Vivek K. Gupta, Rajni Kant

**Affiliations:** a Department of Chemistry, Karnatak Science College, Dharwad 580 001, Karnataka, India; b X-ray Crystallography Laboratory, Postgraduate Department of Physics and Electronics, University of Jammu, Jammu Tawi 180 006, India

## Abstract

In the title compound, C_16_H_16_ClNO_2_S_2_, the piperidine ring is in a chair conformation. In the coumarin ring system, the dihedral angle between the benzene and pyran rings is 3.5 (1)°. In the crystal, a weak C—H⋯O hydrogen bond links mol­ecules into chains along [001]. In addition, π–π stacking inter­actions are present involving the benzene and pyran rings, with a centroid-to-centroid distance of 3.712 (2) Å. The crystal studied is a nonmerohedral twin with refined components 0.221 (1) and 0.779 (1).

## Related literature

For structures and properties of coumarins, see: Kulkarni *et al.* (2006 ▶); Jones *et al.* (1985 ▶); Trenor *et al.* (2004 ▶); Hung *et al.* (2007 ▶). For the applications of dithio­carbamate compounds, see: Bergendorff & Hansson (2002 ▶); Huang *et al.* (2009 ▶). For standard bond lengths, see: Allen *et al.* (1987 ▶). For ring conformations, see: Duax & Norton (1975 ▶). For the synthesis of the title compound, see: Shastri *et al.* (2004 ▶); Vasilliev & Polackov (2000 ▶).
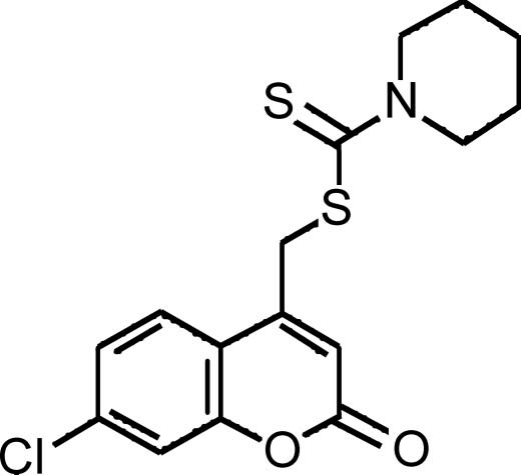


## Experimental

### 

#### Crystal data

C_16_H_16_ClNO_2_S_2_
*M* *_r_* = 353.87
Monoclinic, 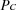

*a* = 4.9427 (3) Å
*b* = 11.5010 (6) Å
*c* = 14.0006 (8) Å
β = 90.271 (6)°
*V* = 795.87 (8) Å^3^
*Z* = 2
Mo *K*α radiation
μ = 0.51 mm^−1^
*T* = 293 K
0.3 × 0.2 × 0.2 mm

#### Data collection

Oxford Xcalibur Sapphire3 diffractometer
Absorption correction: multi-scan (*CrysAlis RED*; Oxford Diffraction, 2010 ▶) *T*_min_ = 0.886, *T*_max_ = 1.000
13944 measured reflections
2801 independent reflections
2678 reflections with *I* > 2σ(*I*)
*R*_int_ = 0.047

#### Refinement

*R*[*F* ^2^ > 2σ(*F* ^2^)] = 0.035
*wR*(*F* ^2^) = 0.082
*S* = 1.04
2801 reflections
200 parameters
2 restraints
H-atom parameters constrained
Δρ_max_ = 0.14 e Å^−3^
Δρ_min_ = −0.18 e Å^−3^
Absolute structure: Flack (1983 ▶), with 1394 Friedel pairs
Flack parameter: −0.01 (10)

### 

Data collection: *CrysAlis PRO* (Oxford Diffraction, 2010 ▶); cell refinement: *CrysAlis PRO* ; data reduction: *CrysAlis RED* (Oxford Diffraction, 2010 ▶); program(s) used to solve structure: *SHELXS97* (Sheldrick, 2008 ▶); program(s) used to refine structure: *SHELXL97* (Sheldrick, 2008 ▶); molecular graphics: *ORTEP-3* (Farrugia, 1997 ▶); software used to prepare material for publication: *PLATON* (Spek, 2009 ▶).

## Supplementary Material

Crystal structure: contains datablock(s) I, global. DOI: 10.1107/S1600536812007933/lh5420sup1.cif

Structure factors: contains datablock(s) I. DOI: 10.1107/S1600536812007933/lh5420Isup2.hkl

Supplementary material file. DOI: 10.1107/S1600536812007933/lh5420Isup3.cml

Additional supplementary materials:  crystallographic information; 3D view; checkCIF report

## Figures and Tables

**Table 1:** Hydrogen-bond geometry (Å, °)

*D*—H⋯*A*	*D*—H	H⋯*A*	*D*⋯*A*	*D*—H⋯*A*
C6—H6⋯O2^i^	0.93	2.41	3.167 (5)	139

Symmetry code: (i) 
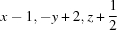
.

**Fig. 1.**
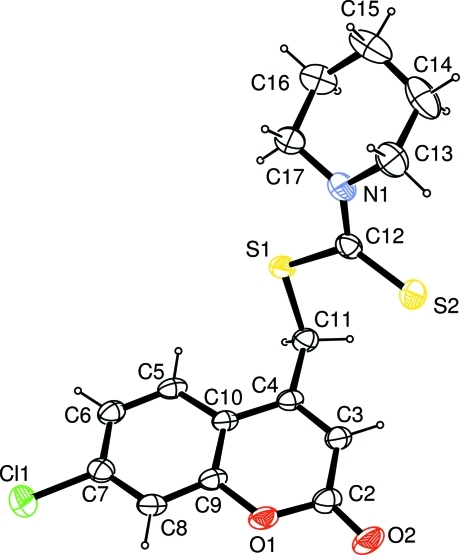
The molecular structure of the title compound. Ellipsoids are drawn at the 40% probability level. H atoms are shown as small spheres of arbitrary radii.

**Fig. 2.**
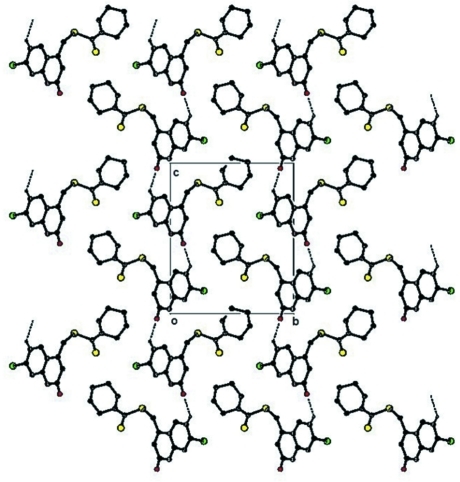
The packing arrangement of molecules viewed along the a axis. The broken lines show the intermolecular C—H···O interactions.

## supplementary crystallographic information

### Comment

Coumarins are an important class of heterocycles, which are widespread in the plant kingdom and have been extensively reported. Coumarin derivatives with various substituents at the C-4 position have revealed potential as anti-microbial, anti-viral, anti-oxidant, anti-inflammatory and anti-cancer agents (Kulkarni *et al.*, 2006). They have also found a place and subsequent use in laser dyes, non-linear optical chromophores, fluorescent whiteners, fluorescent probes and solar energy collectors due to their outstanding optical properties (Jones *et al.*, 1985; Trenor *et al.*, 2004; Hung *et al.*, 2007). Dithiocarbamate (DTC) derivatives are valuable compounds due to their interesting chemistry and utility. These compounds have shown wide applications as pesticides, fungicides in agriculture, sulfur vulcanization and anti-cancer agents (Bergendorff & Hansson, 2002; Huang *et al.*, 2009). In our work, we have been able to link a DTC moiety at C-4 methylene carbon and it was a thought of considerable interest to study the effect of this moiety on the total solid-state conformation of the molecule. A new series of piperidine-1-dithiocarbomate derivatives of 4-substituted coumarin was synthesized in a single step and screened for antimicrobial, anti-diabetic, DNA binding and DNA cleavage activity. In this paper we report the crystal structure of (7-Chloro-2-oxo-2*H*-chromen-4-yl)methyl piperidine-1-carbodithioate (I).

The molecular structure of (I) is shown in Fig. 1. The bond lengths (Allen *et al.*, 1987) and angles in the molecule are within normal ranges. The pyridine ring adopts a normal chair conformation (asymmetry parameters: ΔC_s_(C15—N1) = 0.94; ΔC_2_(C16—C15) = 2.5 (Duax & Norton, 1975). The dihedral angle bewteen pyran and benzene rings in the coumarin moiety 3.5 (1)°. In the crystal, weak C—H···O hydrogen bonds link molecules along [001] (Fig. 2). In addition, π–π interactions between the pyran ring at (*x*, *y*, *z*) and the benzene ring at (1 + *x*, *y*, *z*) are present [centroid separation = 3.712 (2) Å, interplanar spacing = 3.407 Å and centroid shift = 1.47 Å].

### Experimental

4-Bromomethyl coumarin required for the synthesis of the target molecule was synthesized according to an already reported procedure (Shastri *et al.*, 2004) involving the Pechmann cyclization of phenols with 4-Bromoethyl acetoacetate and the potassium salt of piperidine-1-dithiocarbomate was synthesized according to the procedure reported (Vasilliev & Polackov, 2000).

A mixture of 2.73 g (0.01 mol) of 7-chloro-4-bromomethyl coumarin and 1.99 g (0.01 mol) of potassium salt of piperidine-1-dithiocarbomate in 30 ml dry alcohol was stirred for 12 h at room temperature (the reaction was monitored by TLC). The solvent was evaporated and the solid was extracted twice with MDC–water mixture. The organic solvent was dried over CaCl_2_, the solvent evaporated and recrystallized from ethanol–chloroform. A slow evaporation technique was used to grow crystals suitable for diffraction studies in an ethanol–chloroform mixture. Yield = 89%, m.p. 407–409 K. IR (KBr): 1720 cm-1 (C═O), 1430 cm-1 (C═S), 849 cm-1 (C—N), 771 cm-1 (C—Cl). GCMS: m/e: 353.03. 1H NMR (300 MHz, CdCl3, δ, p.p.m.): 2.81 (s, 4H, C13 & C17—H), 2.79 (s, 6H, C14, C16 & C16—H), 4.72 (s, 2H, C4—CH2), 6.21 (s, 1H, C3—H), 7.18 (d, 2H, C6 & C8—H), 7.47 (d, 1H C5—H). Elemental analysis: C, 54.27; H, 4.54; Cl, 10.00; N, 3.92; O, 9.01; S, 18.09.

### Refinement

All H atoms were positioned geometrically and were treated as riding on their parent C atoms, with C—H distances of 0.93–0.97 Å and with *U*_iso_(H) = 1.2U_eq_(C). The crystal studied is a non-merohedral twin with refined components 0.221 (1) and 0.779 (1) and twin law 1.00 0.00 0.00 0.00 1.00 0.00 0.00 0.00 -1.00.

### Crystal data

**Table:**

C_16_H_16_ClNO_2_S_2_	*F*(000) = 368
*M**_r_* = 353.87	*D*_x_ = 1.477 Mg m^−^^3^
Monoclinic, *P**c*	Mo *K*α radiation, λ = 0.71073 Å
Hall symbol: p -2yc	Cell parameters from 7572 reflections
*a* = 4.9427 (3) Å	θ = 3.4–29.0°
*b* = 11.5010 (6) Å	µ = 0.51 mm^−^^1^
*c* = 14.0006 (8) Å	*T* = 293 K
β = 90.271 (6)°	Block, white
*V* = 795.87 (8) Å^3^	0.3 × 0.2 × 0.2 mm
*Z* = 2	

### Data collection

**Table:**

Oxford Xcalibur Sapphire3 diffractometer	2801 independent reflections
Radiation source: fine-focus sealed tube	2678 reflections with *I* > 2σ(*I*)
Graphite monochromator	*R*_int_ = 0.047
Detector resolution: 16.1049 pixels mm^-1^	θ_max_ = 25.0°, θ_min_ = 3.4°
ω scans	*h* = −5→5
Absorption correction: multi-scan (*CrysAlis RED*; Oxford Diffraction, 2010)	*k* = −13→13
*T*_min_ = 0.886, *T*_max_ = 1.000	*l* = −16→16
13944 measured reflections	

### Refinement

**Table:**

Refinement on *F*^2^	Secondary atom site location: difference Fourier map
Least-squares matrix: full	Hydrogen site location: inferred from neighbouring sites
*R*[*F*^2^ > 2σ(*F*^2^)] = 0.035	H-atom parameters constrained
*wR*(*F*^2^) = 0.082	*w* = 1/[σ^2^(*F*_o_^2^) + (0.0367*P*)^2^ + 0.3317*P*] where *P* = (*F*_o_^2^ + 2*F*_c_^2^)/3
*S* = 1.04	(Δ/σ)_max_ = 0.001
2801 reflections	Δρ_max_ = 0.14 e Å^−^^3^
200 parameters	Δρ_min_ = −0.18 e Å^−^^3^
2 restraints	Absolute structure: Flack (1983), with 1394 Friedel pairs
Primary atom site location: structure-invariant direct methods	Flack parameter: −0.01 (10)

### Special details

**Table:**

Experimental. CrysAlisPro, Oxford Diffraction Ltd., Version 1.171.34.40 (release 27–08-2010 CrysAlis171. NET) (compiled Aug 27 2010,11:50:40) Empirical absorption correction using spherical harmonics, implemented in SCALE3 ABSPACK scaling algorithm.
Geometry. All e.s.d.'s (except the e.s.d. in the dihedral angle between two l.s. planes) are estimated using the full covariance matrix. The cell e.s.d.'s are taken into account individually in the estimation of e.s.d.'s in distances, angles and torsion angles; correlations between e.s.d.'s in cell parameters are only used when they are defined by crystal symmetry. An approximate (isotropic) treatment of cell e.s.d.'s is used for estimating e.s.d.'s involving l.s. planes.
Refinement. Refinement of *F*^2^ against ALL reflections. The weighted *R*-factor *wR* and goodness of fit *S* are based on *F*^2^, conventional *R*-factors *R* are based on *F*, with *F* set to zero for negative *F*^2^. The threshold expression of *F*^2^ > σ(*F*^2^) is used only for calculating *R*-factors(gt) *etc*. and is not relevant to the choice of reflections for refinement. *R*-factors based on *F*^2^ are statistically about twice as large as those based on *F*, and *R*-factors based on ALL data will be even larger.

### Fractional atomic coordinates and isotropic or equivalent isotropic displacement parameters (Å^2^)

**Table:**

	*x*	*y*	*z*	*U*_iso_*/*U*_eq_	
S1	0.1990 (2)	0.77560 (7)	0.36977 (7)	0.0421 (3)	
S2	0.4685 (3)	0.60142 (9)	0.24062 (9)	0.0525 (3)	
Cl1	−0.4816 (2)	1.27262 (9)	0.15209 (9)	0.0577 (3)	
O1	0.2148 (6)	0.9922 (2)	0.03114 (17)	0.0429 (7)	
C2	0.4260 (9)	0.9155 (3)	0.0384 (3)	0.0431 (10)	
O2	0.5440 (8)	0.8931 (3)	−0.03402 (19)	0.0583 (9)	
C3	0.4925 (9)	0.8712 (3)	0.1330 (3)	0.0373 (9)	
H3	0.6290	0.8161	0.1393	0.045*	
C4	0.3622 (8)	0.9077 (3)	0.2120 (2)	0.0328 (8)	
C10	0.1525 (8)	0.9952 (3)	0.2026 (2)	0.0315 (8)	
C5	0.0142 (9)	1.0460 (3)	0.2788 (2)	0.0362 (8)	
H5	0.0531	1.0225	0.3410	0.043*	
C6	−0.1802 (9)	1.1309 (3)	0.2633 (3)	0.0405 (10)	
H6	−0.2696	1.1644	0.3147	0.049*	
C7	−0.2409 (8)	1.1657 (3)	0.1707 (3)	0.0372 (9)	
C8	−0.1117 (9)	1.1168 (3)	0.0933 (3)	0.0385 (9)	
H8	−0.1538	1.1396	0.0313	0.046*	
C9	0.0813 (8)	1.0334 (3)	0.1108 (2)	0.0354 (9)	
C11	0.4485 (9)	0.8620 (3)	0.3081 (3)	0.0382 (9)	
H11B	0.4967	0.9276	0.3484	0.046*	
H11A	0.6100	0.8153	0.2998	0.046*	
C12	0.2473 (9)	0.6313 (3)	0.3254 (3)	0.0378 (9)	
N1	0.0894 (7)	0.5538 (3)	0.3692 (3)	0.0487 (9)	
C17	−0.0804 (10)	0.5754 (4)	0.4518 (4)	0.0538 (12)	
H17A	−0.2672	0.5577	0.4360	0.065*	
H17B	−0.0697	0.6569	0.4694	0.065*	
C16	0.0100 (13)	0.5010 (4)	0.5353 (4)	0.0637 (12)	
H16A	−0.1126	0.5127	0.5884	0.076*	
H16B	0.1891	0.5253	0.5557	0.076*	
C15	0.0164 (14)	0.3726 (4)	0.5096 (4)	0.0732 (16)	
H15A	−0.1668	0.3446	0.4997	0.088*	
H15B	0.0962	0.3287	0.5618	0.088*	
C14	0.1810 (12)	0.3540 (4)	0.4192 (4)	0.0719 (17)	
H14B	0.3697	0.3712	0.4323	0.086*	
H14A	0.1684	0.2731	0.4000	0.086*	
C13	0.0825 (11)	0.4303 (3)	0.3383 (4)	0.0609 (14)	
H13A	0.1971	0.4198	0.2829	0.073*	
H13B	−0.1008	0.4087	0.3207	0.073*	

### Atomic displacement parameters (Å^2^)

**Table:**

	*U*^11^	*U*^22^	*U*^33^	*U*^12^	*U*^13^	*U*^23^
S1	0.0626 (7)	0.0293 (5)	0.0344 (5)	0.0036 (5)	0.0134 (5)	0.0018 (4)
S2	0.0701 (7)	0.0446 (6)	0.0429 (5)	0.0132 (5)	0.0119 (6)	−0.0038 (5)
Cl1	0.0600 (7)	0.0446 (6)	0.0686 (8)	0.0118 (5)	0.0082 (6)	0.0067 (5)
O1	0.0627 (18)	0.0443 (16)	0.0218 (12)	0.0010 (14)	0.0080 (13)	−0.0001 (11)
C2	0.064 (3)	0.0350 (19)	0.031 (2)	−0.003 (2)	0.0083 (19)	−0.0062 (16)
O2	0.083 (2)	0.0600 (19)	0.0322 (16)	0.0051 (18)	0.0230 (17)	−0.0058 (13)
C3	0.048 (2)	0.0316 (18)	0.0324 (19)	−0.0030 (18)	0.0071 (19)	−0.0001 (15)
C4	0.041 (2)	0.0289 (18)	0.0287 (19)	−0.0076 (15)	0.0040 (15)	0.0011 (14)
C10	0.040 (2)	0.0283 (18)	0.0258 (19)	−0.0056 (16)	0.0035 (15)	0.0010 (14)
C5	0.050 (2)	0.0362 (19)	0.0224 (17)	−0.0096 (19)	0.0046 (16)	−0.0007 (14)
C6	0.053 (3)	0.034 (2)	0.035 (2)	−0.0039 (18)	0.0123 (18)	−0.0036 (16)
C7	0.040 (2)	0.0297 (19)	0.042 (2)	−0.0062 (16)	0.0032 (18)	0.0033 (16)
C8	0.051 (3)	0.034 (2)	0.0305 (19)	−0.0055 (18)	0.0035 (18)	0.0056 (16)
C9	0.050 (2)	0.0311 (18)	0.0255 (18)	−0.0070 (17)	0.0069 (16)	−0.0014 (14)
C11	0.050 (3)	0.0372 (18)	0.0272 (19)	0.0007 (18)	0.0024 (17)	−0.0029 (16)
C12	0.049 (2)	0.034 (2)	0.0302 (19)	0.0047 (18)	−0.0042 (18)	0.0013 (15)
N1	0.061 (2)	0.0286 (16)	0.056 (2)	0.0022 (15)	0.006 (2)	0.0004 (15)
C17	0.060 (3)	0.037 (2)	0.064 (3)	−0.001 (2)	0.013 (2)	0.009 (2)
C16	0.076 (3)	0.055 (3)	0.060 (3)	−0.005 (3)	−0.005 (3)	0.012 (2)
C15	0.087 (4)	0.044 (2)	0.088 (4)	−0.011 (3)	−0.029 (4)	0.023 (3)
C14	0.070 (4)	0.034 (2)	0.112 (5)	0.001 (2)	−0.028 (4)	−0.003 (3)
C13	0.077 (4)	0.031 (2)	0.075 (3)	−0.003 (2)	0.006 (3)	−0.008 (2)

### Geometric parameters (Å, º)

**Table:**

S1—C12	1.788 (4)	C8—H8	0.9300
S1—C11	1.807 (4)	C11—H11B	0.9700
S2—C12	1.654 (4)	C11—H11A	0.9700
Cl1—C7	1.730 (4)	C12—N1	1.336 (5)
O1—C2	1.371 (5)	N1—C17	1.454 (5)
O1—C9	1.382 (4)	N1—C13	1.485 (5)
C2—O2	1.200 (4)	C17—C16	1.514 (6)
C2—C3	1.456 (5)	C17—H17A	0.9700
C3—C4	1.349 (5)	C17—H17B	0.9700
C3—H3	0.9300	C16—C15	1.521 (7)
C4—C10	1.450 (5)	C16—H16A	0.9700
C4—C11	1.505 (5)	C16—H16B	0.9700
C10—C5	1.398 (5)	C15—C14	1.524 (8)
C10—C9	1.402 (5)	C15—H15A	0.9700
C5—C6	1.386 (6)	C15—H15B	0.9700
C5—H5	0.9300	C14—C13	1.511 (7)
C6—C7	1.388 (5)	C14—H14B	0.9700
C6—H6	0.9300	C14—H14A	0.9700
C7—C8	1.380 (6)	C13—H13A	0.9700
C8—C9	1.374 (6)	C13—H13B	0.9700
			
C12—S1—C11	104.60 (18)	N1—C12—S1	112.4 (3)
C2—O1—C9	121.8 (3)	S2—C12—S1	122.2 (2)
O2—C2—O1	116.7 (4)	C12—N1—C17	126.3 (3)
O2—C2—C3	125.9 (4)	C12—N1—C13	121.2 (4)
O1—C2—C3	117.5 (3)	C17—N1—C13	112.5 (4)
C4—C3—C2	122.1 (4)	N1—C17—C16	110.4 (4)
C4—C3—H3	119.0	N1—C17—H17A	109.6
C2—C3—H3	119.0	C16—C17—H17A	109.6
C3—C4—C10	119.1 (3)	N1—C17—H17B	109.6
C3—C4—C11	119.3 (4)	C16—C17—H17B	109.6
C10—C4—C11	121.5 (3)	H17A—C17—H17B	108.1
C5—C10—C9	116.6 (3)	C17—C16—C15	111.8 (5)
C5—C10—C4	125.0 (3)	C17—C16—H16A	109.2
C9—C10—C4	118.5 (3)	C15—C16—H16A	109.2
C6—C5—C10	121.0 (3)	C17—C16—H16B	109.2
C6—C5—H5	119.5	C15—C16—H16B	109.2
C10—C5—H5	119.5	H16A—C16—H16B	107.9
C5—C6—C7	119.8 (4)	C16—C15—C14	110.2 (4)
C5—C6—H6	120.1	C16—C15—H15A	109.6
C7—C6—H6	120.1	C14—C15—H15A	109.6
C8—C7—C6	121.1 (4)	C16—C15—H15B	109.6
C8—C7—Cl1	119.5 (3)	C14—C15—H15B	109.6
C6—C7—Cl1	119.4 (3)	H15A—C15—H15B	108.1
C9—C8—C7	117.9 (4)	C13—C14—C15	111.7 (5)
C9—C8—H8	121.0	C13—C14—H14B	109.3
C7—C8—H8	121.0	C15—C14—H14B	109.3
C8—C9—O1	115.4 (3)	C13—C14—H14A	109.3
C8—C9—C10	123.6 (3)	C15—C14—H14A	109.3
O1—C9—C10	120.9 (3)	H14B—C14—H14A	107.9
C4—C11—S1	115.4 (3)	N1—C13—C14	109.3 (4)
C4—C11—H11B	108.4	N1—C13—H13A	109.8
S1—C11—H11B	108.4	C14—C13—H13A	109.8
C4—C11—H11A	108.4	N1—C13—H13B	109.8
S1—C11—H11A	108.4	C14—C13—H13B	109.8
H11B—C11—H11A	107.5	H13A—C13—H13B	108.3
N1—C12—S2	125.5 (3)		
			
C9—O1—C2—O2	−173.5 (4)	C5—C10—C9—C8	0.5 (5)
C9—O1—C2—C3	4.7 (5)	C4—C10—C9—C8	−179.1 (4)
O2—C2—C3—C4	174.8 (4)	C5—C10—C9—O1	177.1 (3)
O1—C2—C3—C4	−3.2 (6)	C4—C10—C9—O1	−2.5 (5)
C2—C3—C4—C10	−1.1 (6)	C3—C4—C11—S1	−115.6 (4)
C2—C3—C4—C11	−177.6 (4)	C10—C4—C11—S1	68.0 (4)
C3—C4—C10—C5	−175.7 (4)	C12—S1—C11—C4	86.3 (3)
C11—C4—C10—C5	0.7 (5)	C11—S1—C12—N1	175.5 (3)
C3—C4—C10—C9	3.9 (5)	C11—S1—C12—S2	−3.6 (3)
C11—C4—C10—C9	−179.7 (3)	S2—C12—N1—C17	171.5 (4)
C9—C10—C5—C6	−0.9 (5)	S1—C12—N1—C17	−7.6 (5)
C4—C10—C5—C6	178.7 (4)	S2—C12—N1—C13	−5.4 (6)
C10—C5—C6—C7	0.5 (6)	S1—C12—N1—C13	175.4 (3)
C5—C6—C7—C8	0.3 (6)	C12—N1—C17—C16	−117.9 (5)
C5—C6—C7—Cl1	−179.4 (3)	C13—N1—C17—C16	59.3 (5)
C6—C7—C8—C9	−0.7 (6)	N1—C17—C16—C15	−55.2 (6)
Cl1—C7—C8—C9	179.0 (3)	C17—C16—C15—C14	52.2 (7)
C7—C8—C9—O1	−176.5 (3)	C16—C15—C14—C13	−53.3 (6)
C7—C8—C9—C10	0.3 (6)	C12—N1—C13—C14	117.5 (5)
C2—O1—C9—C8	175.0 (3)	C17—N1—C13—C14	−59.8 (6)
C2—O1—C9—C10	−1.9 (5)	C15—C14—C13—N1	56.3 (6)

### Hydrogen-bond geometry (Å, º)

**Table:**

*D*—H···*A*	*D*—H	H···*A*	*D*···*A*	*D*—H···*A*
C17—H17*B*···S1	0.97	2.36	2.923 (5)	116
C13—H13*A*···S2	0.97	2.55	3.067 (4)	113
C6—H6···O2^i^	0.93	2.41	3.167 (5)	139

Symmetry code: (i) *x*−1, −*y*+2, *z*+1/2.
